# Robust Heterojunctions of Metallic Alloy and Carbon Fiber-Reinforced Composite Induced by Laser Processing

**DOI:** 10.3390/ma14237469

**Published:** 2021-12-06

**Authors:** Haipeng Wang, Peng Yan, Yingchun Guan

**Affiliations:** 1 Key Laboratory of High-Efficiency and Clean Mechanical Manufacture of MOE, School of Mechanical Engineering, Shandong University, Jinan 250061, China; wang_haipeng@sdu.edu.cn; 2 School of Mechanical Engineering and Automation, Beihang University, Beijing 100083, China; 3 Ningbo Institute of Technology, Beihang University, Ningbo 315800, China

**Keywords:** laser joining, heterojunction, fracture strength, structure density, failure mechanism

## Abstract

The development of heterojunctions with a strong bonding interface between metals and non-metals has attracted much attention owing to their great potential for use in lightweight structures. Laser joining technology, which emerged as a fast and reliable method, has proven its feasibility and unique advantages in joining metal to polymer matrix composites. Herein, an optimized laser joining configuration has been employed to realize high-quality joining of titanium alloy and carbon fiber-reinforced composite. Cross-sectional microstructures of laser-produced joints reveal that micro-bubbles near the interface have been effectively suppressed and eliminated due to the continual clamping pressure applied to the joined area during the joining process. Tensile tests suggest that the joint strength increases with structure density on a titanium alloy surface, and the greatest fracture strength of joints reaches more than 60 MPa even after experiencing a high–low temperature alternating aging test. For higher structure density (>95%), the joints fail by the fracture of parent plastics near the joined area due to the tensile-loading-induced peel stress at the edges of the overlap region. Otherwise, the joints fail by interfacial shear fracture with breakage when the structure density is lower than 91.5%. The obtained high-performance heterojunctions show great potential in the aerospace and automotive fields.

## 1. Introduction

The manufacture of lightweight materials has always been one of the goals pursued in advanced manufacturing industries such as automotive engineering and aerospace. Towards this goal, various light materials including carbon fiber- and glass fiber-reinforced thermoplastics (CFRTP/GFRTP) have been increasingly utilized to replace metals due to their low density, high specific strength and good chemical resistance [1,2,3,4]. In practical applications of CFRTP/GFRTP, it is inevitable to confront the issue of achieving high-strength heterojunctions between metals and thermoplastic composites. In view of such demand, various strategies for joining metals and thermoplastics have been developed in past decades such as adhesive bonding [5,6], mechanical fastening [7,8], and thermal welding [9,10,11]. In comparison with adhesive bonding or mechanical fastening methods, thermal welding technologies such as laser-assisted joining (LAJ) relied on a thermal conduction process and produced joints showing better environmental adaptability and more homogeneous stress distribution [12,13]. The LAJ process has several advantages: no contact is required; it has high flexibility and enables precise processing; and it can be flexibly adjusted (e.g., laser parameters, LAJ configuration) to meet the joining requirements between dissimilar specimens with specific characteristics (e.g., material, dimension, shape). For example, in the FlexHyJoin project funded by the European Union’s Horizon 2020 research and innovation program, the LAJ process was successfully employed to join the metal bracket and composite rib on the Fiat city car [14].

The construction of mechanical interlocks between metal and polymer matrix composites is the commonly used strategy to improve joint strength. Jiao et al. reported a hybrid surface pre-treating method undertaken by machining micro-textures on an Al alloy surface and adding a PA layer at the CFRTP-Al alloy interface to enhance the CFRP-Al joint strength [15]. Thanks to the increased contact area and the mechanical interlocks generated between the CFRTP and Al alloy, the maximum joint strength reached 37.5 MPa. During the laser joining process, the widespread generation of porosity defects near the bonding interface remains a challenge hindering the further improvement of joint strength. Shan et al. revealed formation mechanisms of the porosity in CFRTP, and suppressed shrinkage porosity by fabricating protrusions on steel to change the heat conduction path during the LAJ process and form the mechanical interlocks at the joining interface [16]. The fracture strength of the resultant joints was increased from 9.3 MPa to 30.8 MPa. Arkhurst et al. stated that the generated porous oxide layer on Mg alloy was beneficial to the formation of micro-mechanical interlocks and the suppression of porosity defects at the joining interface [11]. Chen et al. introduced the use of an ultrasonic transducer tool in the laser joining process to remove the laser-induced bubbles from the bonding zone between metal and polyethylene terephthalate (PET) based on the ultrasonic vibration-induced pressure on the sample [17]. The resultant joint strength was enhanced from 10 MPa to more than 40 MPa due to the reduction in bubbles. However, the aforementioned methods present low universality and poor controllability.

In this study, we accordingly proposed a facile optimized laser joining configuration to suppress the porosity near the joining interface as well as construct strong mechanical interlocks between titanium alloy and carbon fiber-reinforced polyetheretherketone (CF30/PEEK). The mechanism for suppressing porosity defects lies in the mechanical squeezing pressure continuously applied to the joined area during the laser joining process, where the laser-induced bubbles are squeezed out from the molten zone due to the flow of melted plastics. Heterojunctions with a high bonding strength of over 60 MPa were achieved in this study and remained even after long-term high–low temperature alternating aging tests from −25 to 125 °C.

## 2. Materials and Methods

### 2.1. Preparation of Specimens

**Figure 1:**
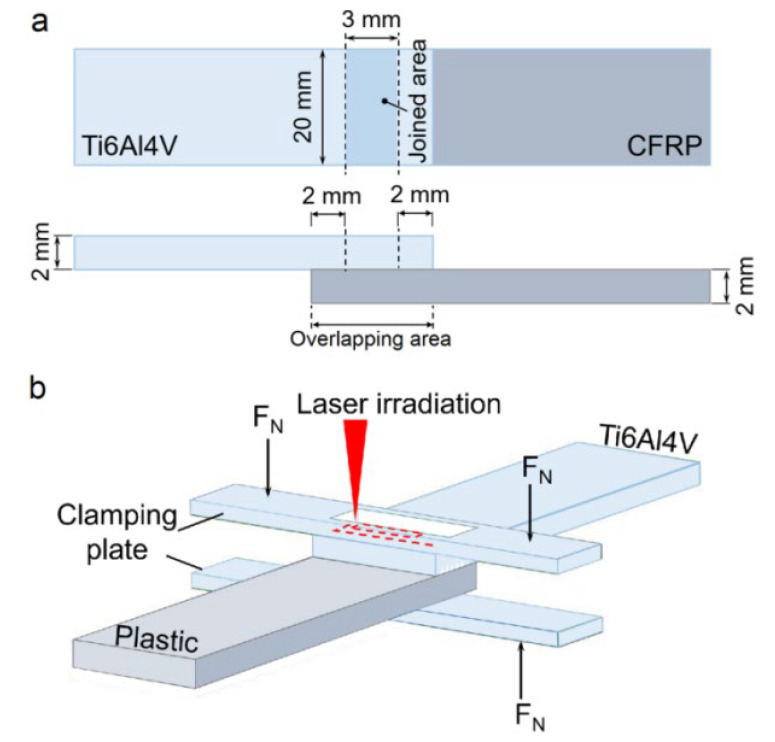
Schematic diagrams showing the joined area (**a**) and the optimized laser joining configuration (**b**).

The utilized polymer is a commercial CF30/PEEK laminate composed of 30% short carbon fibers and 70% polyetheretherketone matrix, showing a tensile strength of ~210 MPa at 23 °C (ISO 527). A commercial Ti6Al4V plate is used as the metal part. The specimen dimensions of both CF30/PEEK and Ti6Al4V are 20 mm × 100 mm × 2 mm. Micro-texturing pre-treatments are undertaken on the joined areas of Ti6Al4V surfaces via an ultrafast laser system (Time Bandwidth; Duetto, Light Conversion, Lithuania) which produces 209 fs pulses at a repetition rate of 100 kHz with a central wavelength of 1030 nm. The laser beam has a Gaussian profile with a TEM00 (M^2^ < 1.3) spatial mode, and is directed to the sample surface using a galvanometric scanner with a telecentric f-theta lens. The focal length is 85 mm. Therefore, “V-shape” microgrooves with finely tunable depth and width of, respectively, 500 ± 50 μm and 255 ± 35 μm were fabricated on Ti6Al4V surfaces via laser irradiating along the designed parallel and isometric laser scanning lines. Regular micropillar arrays were prepared by laser irradiating on titanium alloy surfaces along two perpendicular directions. The pitch between two adjacent microgrooves varied from 300 μm to 350 μm. Structure density on the metal surface, defined as the ratio of the projected area of microstructure to the joined area, is a critical factor influencing the joint quality at the joining interface. The structure density of the microgrooves on the Ti6Al4V is calculated as follows:

(1) $$S_{df}=\frac{w_{g}\times b\times N}{a\times b}$$

where *w*_g_, *a*, *b,* and *N* are the width of microgrooves, the length and width of the joined area, and the number of microgrooves, respectively. The structure density of the micropillars on Ti6Al4V is calculated as follows,

(2) $$S_{dp}=\frac{w_{g}\times \left(a\times N_{a}+b\times N_{b}-w_{g}\times N_{a}\times N_{b}\right)}{a\times b}$$

where *N_a_* and *N_b_* are, respectively, the number of microgrooves along the length and width directions of the joined area. Thus, the structure density on Ti6Al4V can be tuned by varying the pitch and width of the microgrooves. The joined area between Ti6Al4V and CF30/PEEK is set to be 3 mm × 20 mm (Figure 1a). The optimized laser parameters for machining micro-structures are summarized in Table 1. After laser pre-treatment, the prepared specimens were ultrasonically cleaned for 30 min using deionized water.

### 2.2. Laser Joining Procedure

Before the laser joining process, the titanium alloy plate with a micro-structured surface was placed on the CF30/PEEK with the structured surface in contact with it. The overlapping area between Ti6Al4V and CF30/PEEK was set to be slightly larger than the joined area as illustrated in Figure 1a. Two preset Ti6Al4V clamping plates perpendicular to the overlapping direction were used to provide suitable compressive pressure for the overlapping area, as shown in Figure 1b. The exerted pressure on the clamping plate was about 460 N. It is worth noting that the materials in the center of the upper clamping plate were cut off to generate a rectangular hole with dimensions of 4 mm × 21 mm, so that the laser beam was directly irradiated on the surface of the joined titanium alloy through the hole, as illustrated in Figure 1b.

The laser joining process was carried out using a continuous laser scanning system on the focal plane. The processing parameters used for joining are listed in Table 2. In the laser joining process, the laser energy is absorbed by the Ti6Al4V surface, and the heat is generated in Ti6Al4V and conducts through it to heat the Ti6Al4V–CF30/PEEK interface. When the composites near the interface are heated to reach the melted state, the melted composites will be forced to flow and fill the microgrooves under the clamping pressure. As the laser beam moves away from the irradiated surface, the melted composites are cooled and consolidated, resulting in the generation of mechanical interlocks at the joining interface.

### 2.3. Property Characterization

Surface morphologies of the laser-fabricated microstructures on the titanium alloy surface and the fractured surfaces of the joints are characterized by scanning electron microscopy (SEM, JSM-6610LV, JEOL, Peabody, MA, USA). After the laser joining process, cross-sectional microstructures near the joining interface were characterized using SEM. The strength of the LAJ-produced joint between Ti6Al4V and CF30/PEEK was tested by an electronic universal testing machine (Instron 5982, Instron, Boston, MA, USA). Two pads of the same thickness as the CCF30/PEEK and Ti6Al4V plates were tabled on both ends of the sample for alignment during the test. The tests were carried out at a traveling speed of 1 mm/min. Five samples in each joining condition were prepared for shear strength testing to reduce the experimental error.

## 3. Results and Discussion

**Figure 2:**
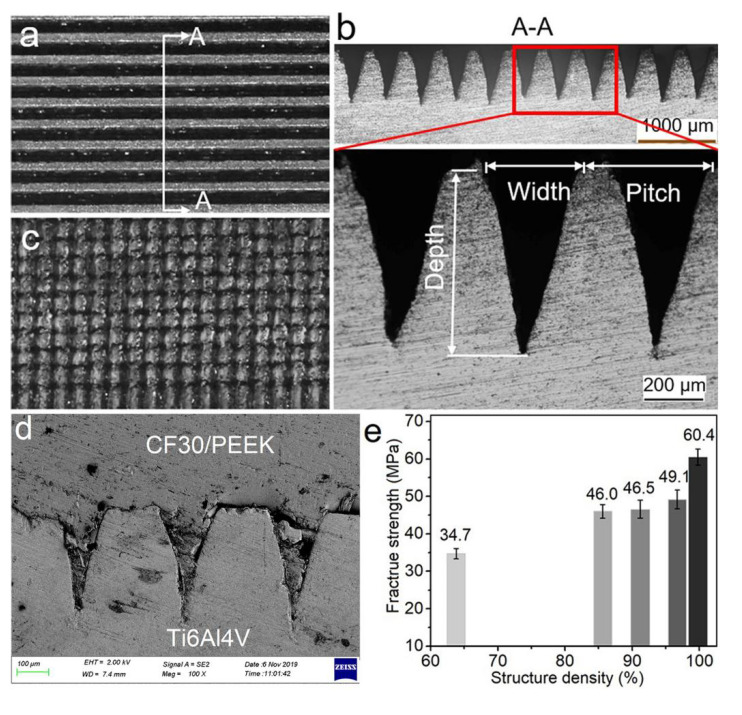
(**a**–**c**) Surface morphology and cross-sectional microstructure of laser-fabricated microgrooves (*S_df_* = 63.8%), (**a**,**b**) and micropillars (*S_dp_* = 85.6%, (**c**) on Ti6Al4V surface. (**d**) Typical cross-sectional microstructures of LAJ-produced Ti6Al4V-CF30/PEEK joints (*S_df_* = 63.8%). (**e**) Fracture strength of LAJ-produced Ti6Al4V-CF30/PEEK joints with increasing structure density.

Figure 2a–c show surface morphologies and cross-sectional microstructures of the laser-fabricated microgrooves and micropillars on the Ti6Al4V surface. Benefiting from the high-precision characteristics of femtosecond laser micromachining technology, both the structure dimension and the shape of the generated microgrooves and micropillars present high uniformity, as exhibited by the cross-sectional structures in Figure 2b. By tuning the width of the microgrooves from 225 μm to 290 μm as well as the pitch between two adjacent microgrooves from 300 μm to 350 μm, regular microgroove or micropillar arrays with structure densities of 63.8%, 85.6%, 91.2%, 96.7%, and 99.8%, respectively, were obtained (Table 3). After the laser joining process, the cross-sectional microstructures of the produced Ti6Al4V-CF30/PEEK joints revealed that the melted plastic composites had reached the bottom of microgrooves, and all the microgrooves or the gaps between micropillars had been completely filled by CF30/PEEK composites (Figure 2d), independent of the structure density on the Ti6Al4V surface. Therefore, superior mechanical interlocks form between Ti6Al4V and CF30/PEEK, despite the high viscosity and low flowability of the melted PEEK [18]. This mainly benefits from the pre-exerted clamping pressure on the joining area (Figure 1b). During laser irradiating, when the temperature of the PEEK matrix near the interface increases to the melt point, the melt of the PEEK matrix occurs, then the melted polymer matrix, together with the short carbon fibers blended in it, are forced to flow and fill the microgrooves or the gaps between micropillars under the clamping pressure. This process is accompanied by the air among the microstructures being pushed out. The strong mechanical interlocks formed near the interface greatly contribute to the joint strength. As presented in Figure 2e, the fracture strength of the joints is improved from 34.7 MPa to 46.5 MPa as the structure density increases from 63.8% to 91.2%, and the greatest fracture strength reaches 60.4 MPa, which is significantly higher than previously reported results (<45 MPa) [16,17,19,20].

**Figure 3:**
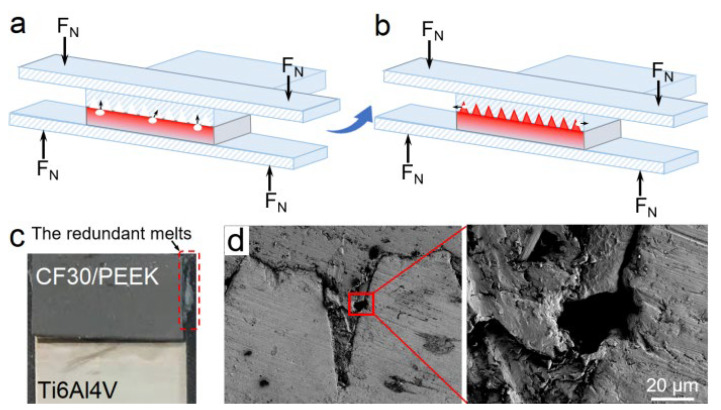
(**a**,**b**) Schematic diagrams showing the movement of the PEEK pyrosis-produced bubbles during the laser joining process. (**c**) Spill-out of the redundant melts after the laser joining process. (**d**) Cross-sectional microstructures of typical Ti6Al4V-CF30/PEEK joints (*S_df_* = 63.8%) with few microbubbles remaining near the interface.

In the laser joining process, pyrolysis of molten PEEK is prone to occur near the interface due to high temperature distributions, leading to the generation of gas bubbles, as illustrated in Figure 3a,b [21,22]. These bubbles will be squeezed by surrounding melts and forced to flow under the clamping pressure; near the interface, some of these are squeezed out from the melts. When the gaps between microstructures are completely filled, a small amount of redundant melts are squeezed out from the joining interface (Figure 3c), which also takes away some bubbles. Moreover, the continual clamping pressure exerted on the joined area makes it easy to avoid the shrinkage porosity commonly generated far from the interface during the solidification of the melt [16,23]. After optimizing the laser joining process by controlling the process duration and the temperature evolution near the interface, only a few microbubbles remain near the interface of the produced joints (Figure 3d), which has little effect on the joint strength.

**Figure 4:**
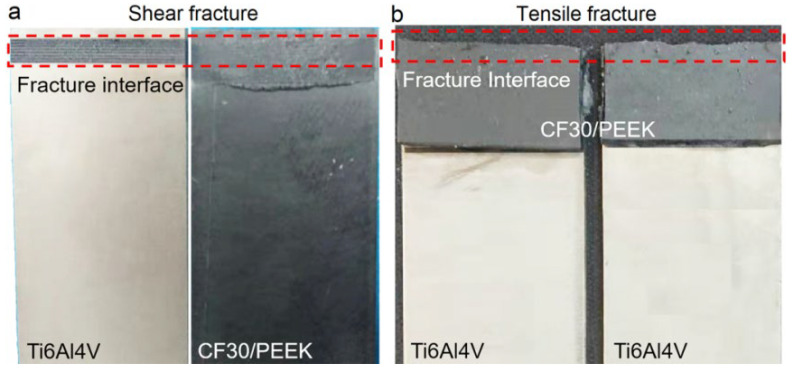
Two typical failure modes of the LAJ-produced Ti6Al4V-CF30/PEEK joints during tensile tests. (**a**) Shear fracture along metal–plastic interface (*S_dp_* = 91.2%). (**b**) Tensile fracture of parent plastic material (*S_dp_* = 99.8%).

**Figure 5:**
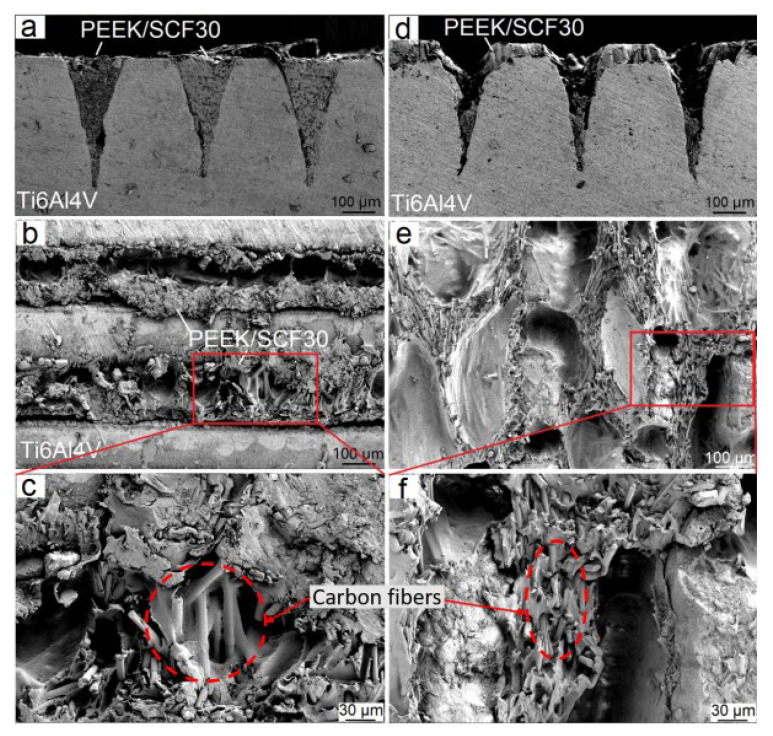
Typical cross-sectional images of the fractured joints ((**a**), (*S_df_* = 63.8%), (**d**), (*S_dp_* = 85.6%)) and the morphologies of the detached surfaces (**b**,**c**,**e**,**f**) after tensile tests.

Figure 4 shows two typical fracture modes of joints with different structure densities during tensile tests. The joints with a structure density below 91.5% failed by the separation along the metal–plastic interface, whereas the joints with a structure density above 95% failed by the fracture of parent plastic material near the joined area. The former failure mode involves an interfacial shear fracture with CF30/PEEK breakage, which is manifested by morphological and topographical features of the detached surfaces after tensile tests (Figure 5). Figure 5a,b,d,e shows that the plastics inserted inside microgrooves or gaps between micropillars remain in the structures after tensile tests, and the interfacial fracture occurs by the shear tearing of the CF30/PEEK composite along the metal–plastic interface. From Figure 5c,f it can be seen that the remaining plastics on the detached surfaces are blended with numerous short carbon fibers. This further demonstrates that short carbon fibers have filled in the microgrooves or the gaps between micropillars with the flow of melts during the laser joining process, so that the carbon fiber as a reinforcing phase in matrix does indeed play a role in enhancing the bonding performance of joints.

**Figure 6:**
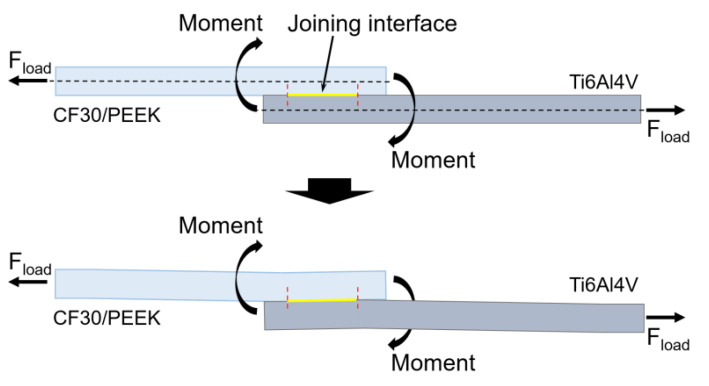
Schematic diagram showing the bending moment induced by eccentric loading in a single-lap joint subject to tensile loading.

As the structure density increased to 96.7% (microgrooves) and 99.8% (micropillars) by decreasing the pitch between adjacent microgrooves or micropillars to 300 μm and increasing the width of microgrooves to 290 μm, the joints failed by the fracture of parent plastic material near the joined area (Figure 4b). On the one hand, the higher structure density leads to a stronger bonding performance of the joints at the metal–plastic interface, thus it becomes more difficult for the CF30/PEEK composite to fracture along the metal–plastic interface. On the other hand, when single-lap joints are subjected to tensile loading, the force lines in the joined parts do not overlap with the joining line (Figure 6), leading to a bending moment in the joint [24,25]. The eccentric loading-induced bending moment results in the generation of peel stress at the edges of the overlap region [17,26]. When the peel stress reaches the fracture stress of any joined part, fracture occurs near the overlap region (Figure 4b).

**Figure 7:**
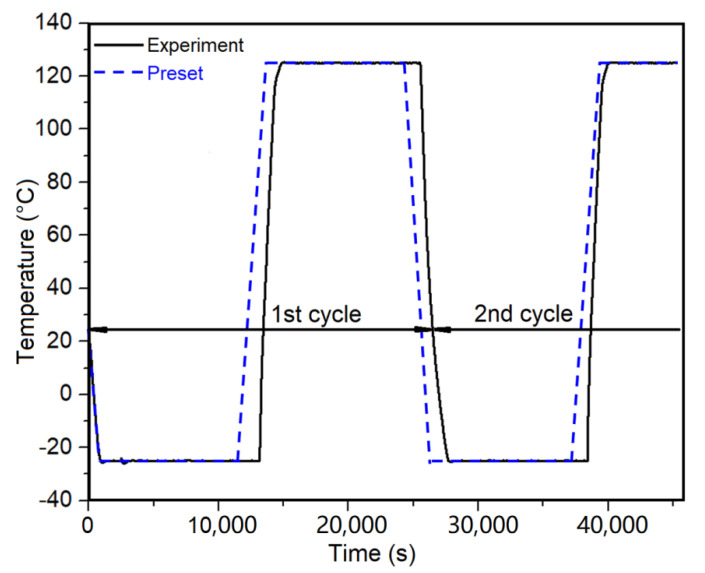
The preset and experimental temperature variation curves in climatic chamber during the high-low temperature alternating aging tests.

**Figure 8:**
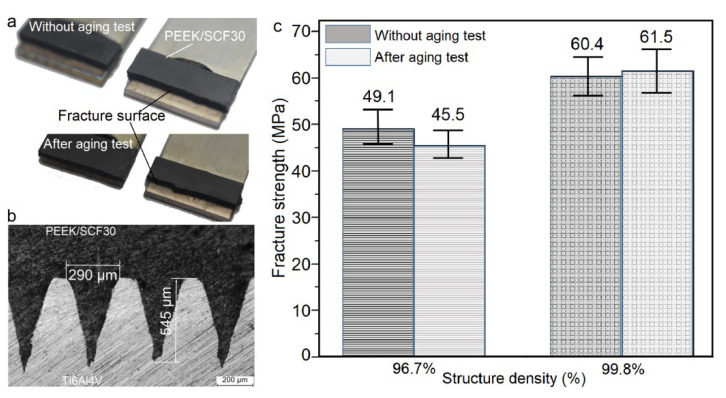
(**a**) The failure of Ti6Al4V-CF30/PEEK joints before and after the aging test. (**b**) The typical cross-sectional microstructures of the joint after the aging test. (**c**) Fracture strength of the LAJ-produced Ti6Al4V-CF30/PEEK joints before and after high–low temperature alternating aging tests.

Considering the engineering applications of joints under complex environmental conditions, the produced Ti6Al4V-CF30/PEEK joints with structure densities of 96.7% and 99.8% were selected to conduct high–low temperature alternating aging tests from −25 to 125 °C for 20 cycles, with each cycle lasting 7 hours. The preset and experimental temperature variation curves in the climatic chamber during the aging tests are shown in Figure 7. The long-term temperature variations resulted in the generation and accumulation of thermal stress at the joint interface, owing to the different thermal expansion coefficients of CF30/PEEK and titanium alloy [27]. The accumulated thermal stress may lead to the generation and enlargement of microcracks near the interface, finally deteriorating the joint strength. Results from tensile tests revealed that after the aging test, the joints failed by the fracture of the CF30/PEEK near the joined area (Figure 8a). Similar to the cross-sectional microstructures of the joints without the aging treatment (Figure 2d), no obvious porosity or microcrack was observed near the joint interface after the aging test (Figure 8b). Moreover, the fracture strength of the joints before and after the aging test changed little, as shown in Figure 8c. These results experimentally suggest that the thermal stress induced by the aging test near the joint interface has little effect on the joint strength. As a consequence, the LAJ-produced Ti6Al4V-CF30/PEEK joints present high bonding strength and good anti-aging properties.

## 4. Conclusions

In summary, an optimized laser joining process was utilized to join titanium alloy and CF30/PEEK by exerting continual clamping pressure on the joined area using two preset plates perpendicular to the overlapping direction. The Ti6Al4V-CF30/PEEK joints with a strong bonding interface were achieved due to the construction of mechanical interlocks as well as the avoidance or elimination of porosity near the joint interface. The obtained joints present high fracture strength and excellent anti-aging properties with the largest fracture strength reaching over 60 MPa. Correspondingly, the joints failed by the fracture of the parent CF30/PEEK near the joined area due to the eccentric-loading-induced bending moment at the edges of the overlap region during tensile tests. The realization of strong bonding performance between titanium alloy and CF30/PEEK shows great potential in lightweight engineering applications in fields such as aviation and the automotive industry.

## Figures and Tables

**Table 1:** The laser parameters for machining micro-structures on the Ti6Al4V surface.

Laser Power	Pulse Duration	Frequency	Scanning Speed	Beam Diameter
15 W	209 fs	100 kHz	500 mm/s	~35 μm

**Table 2:** The laser parameters used for joining Ti6Al4V–CF30/PEEK.

Laser Type	Laser Power	Wavelength	Scanning Speed	Beam Diameter	Focal Length
Continuous fiber laser	200 W	1030 nm	2000 mm/s	~30 μm	374 mm

**Table 3:** The parameters of the laser-fabricated micro-structures on the Ti6Al4V surface.

Case	Microgroove Width (*w_g_*), μm	Pitch, μm	Texture	Structure Density, %
1	225	350	Groove	63.8
2	225	350	Pillar	85.6
3	255	350	Pillar	91.2
4	290	300	Groove	96.7
5	290	300	Pillar	99.8

## Data Availability

The datasets used or analyzed during the current study are available from the corresponding author on reasonable request.
